# Deciphering Human Leukocyte Antigen Susceptibility Maps From Immunopeptidomics Characterization in Oncology and Infections

**DOI:** 10.3389/fcimb.2021.642583

**Published:** 2021-05-28

**Authors:** Pablo Juanes-Velasco, Alicia Landeira-Viñuela, Vanessa Acebes-Fernandez, Ángela-Patricia Hernández, Marina L. Garcia-Vaquero, Carlota Arias-Hidalgo, Halin Bareke, Enrique Montalvillo, Rafael Gongora, Manuel Fuentes

**Affiliations:** ^1^ Department of Medicine and Cytometry General Service-Nucleus, CIBERONC, Cancer Research Centre (IBMCC/CSIC/USAL/IBSAL), Salamanca, Spain; ^2^ Proteomics Unit, Cancer Research Centre (IBMCC/CSIC/USAL/IBSAL), Salamanca, Spain

**Keywords:** immunopeptidomics, human leukocyte antigen, immunochromatography, proteomics, vaccines

## Abstract

Genetic variability across the three major histocompatibility complex (MHC) class I genes (human leukocyte antigen [HLA] A, B, and C) may affect susceptibility to many diseases such as cancer, auto-immune or infectious diseases. Individual genetic variation may help to explain different immune responses to microorganisms across a population. HLA typing can be fast and inexpensive; however, deciphering peptides loaded on MHC-I and II which are presented to T cells, require the design and development of high-sensitivity methodological approaches and subsequently databases. Hence, these novel strategies and databases could help in the generation of vaccines using these potential immunogenic peptides and in identifying high-risk HLA types to be prioritized for vaccination programs. Herein, the recent developments and approaches, in this field, focusing on the identification of immunogenic peptides have been reviewed and the next steps to promote their translation into biomedical and clinical practice are discussed.

## Introduction

Immunopeptidome is known as the list of peptides (independent of length, short-large list) presented on the surface of the cell by class I and class II human leukocyte antigen (HLA) molecules, which activate the immune response through selective and specific recognition by T cells. Currently, immunopeptidomes is gaining high significance in basic and translational biomedical science. Regarding basic research, to understand the immune system and specific responses to tolerogenic and non-tolerogenic antigenic stimulus, an exhaustive analysis of the immunopeptidome could be highly relevant and a key point to understand the mechanisms of immune response to be able to manipulate the specific immune responses. About translational biomedical research, the accurate knowledge of the immunopeptidome could improve immunotherapies and help in the next generation vaccine development against cancer, autoimmune or infectious diseases (Human Immuno-Peptidome Project, 2015; Mahdi, 2019). Bearing these concepts in mind, deciphering the immunopeptidome is becoming of great interest.

The HLA system is a group of proteins encoded by the major histocompatibility complex (MHC) genes and they present peptides (antigens) to T lymphocytes. They are expressed on the cell membrane, and Class I molecules are displayed on all human cells (except for red blood cells). The principal function of these cell surface proteins is the regulation of the adaptive immune responses by engaging with the cognate T cell receptor. They are also important for the self vs. nonself discrimination by the immune system for distinguishing between the body’s own proteins and foreign proteins from invaders (such as viruses, bacteria, or any type of pathogen). Moreover, the HLA system is also involved in the immunopathogenesis of many diseases, such as oncology and autoimmune pathologies, among others (Human Immuno-Peptidome Project, 2015; Mahdi, 2019).

The HLA system is composed of genes (all of them are encoded in chromosome 6) that are co-dominantly expressed and highly polymorphic. The HLA molecules are classified into two main classes, which are: *i.*-MHC class I complex is composed of major genes (HLA-A, HLA-B, HLA-C) and non-classical genes (HLA-E, HLA-F, HLA-G). The function of the MHC class I complex is the presentation of intracellular peptides to CD8 + cytotoxic T lymphocytes. *ii.*- MHC class II complex is composed of major genes (HLA-DP, HLA-DQ, HLA-DR) and non-classical genes (HLA-DM, HLA-DO). The function of the MHC class II complex is to present extracellular processed antigenic peptides to CD4+ helper T cells (Human Immuno-Peptidome Project, 2015; Mahdi, 2019).

**Figure 1:**
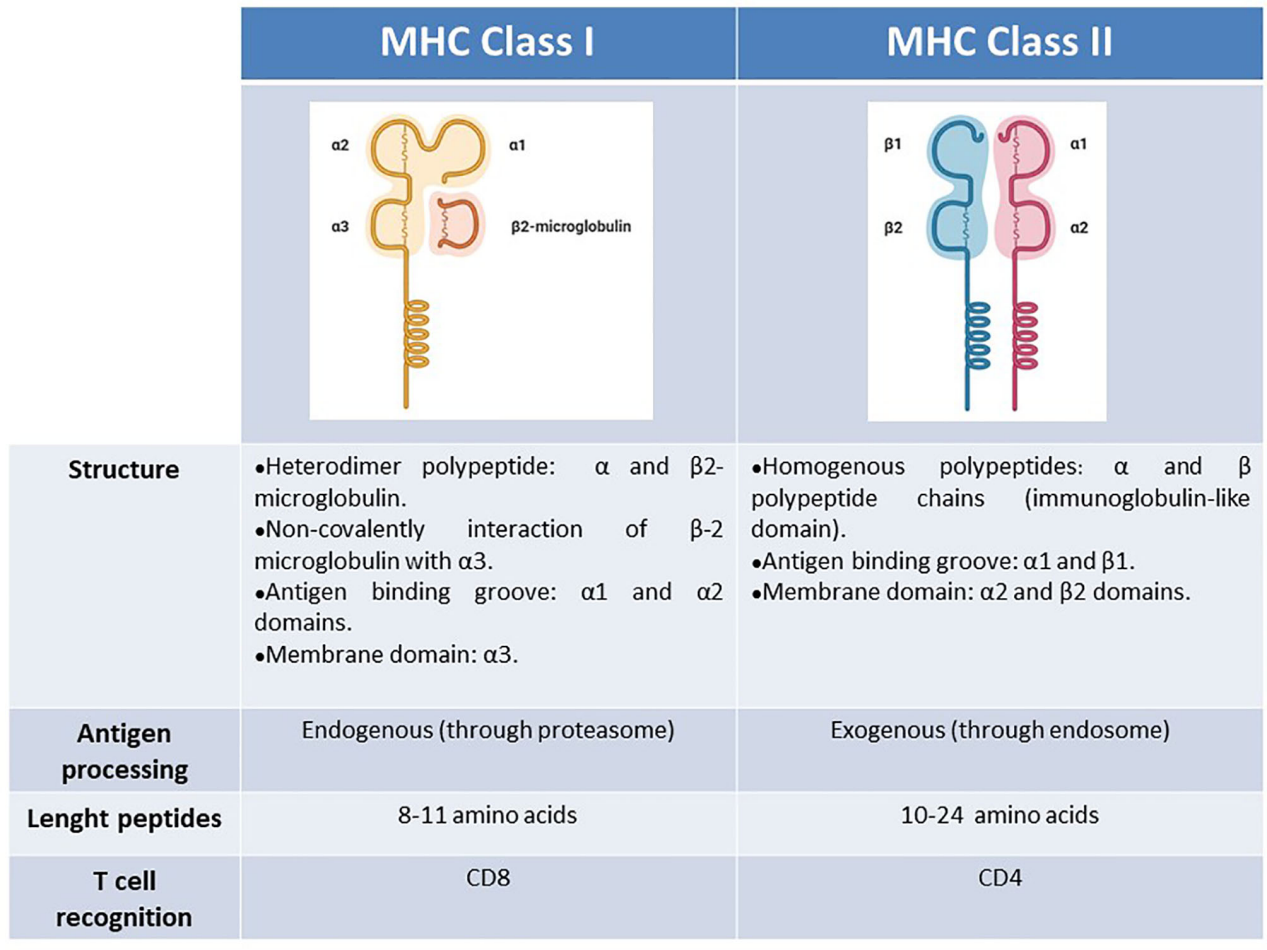
Schematic summary of MHC characteristics. Structure, antigen processing, peptides length, T-cell recognition.

As the source of the peptide is different between these two classes, MHC class I and II molecules have different intracellular pathways for antigen processing. This difference ranges from the HLA complex formation to the peptide loading and HLA migration to cell membrane. Therefore, the structural properties of the epitopes presented on these classes differ from each other. In HLA Class I antigen processing, the first step takes place in the cytosol where intracellular antigens are degraded, mainly by proteolysis. Then, the antigen precursor peptides are transported to the endoplasmic reticulum, where they are further modified into short linear peptides, 8 to 11 amino acids (aa´) which are assembled with HLA class I. This class I HLA-peptide complex go out to the surface of the cell and is carefully checked by CD8 + T cells for foreign antigens that differ from healthy or normal ones (Human Immuno-Peptidome Project, 2015; Mahdi, 2019; Purcell et al., 2019). When CD8+ T cells recognize the presented antigens through their T cell receptors, they ultimately eliminate the affected cells through the cytotoxic arsenal of these effector immune cells. On the other hand, the exogenous antigen will be recognized and captured by a professional antigen presenting cell (APC). Then, exogenous antigens are degraded at the endosomal compartment of the APC and the resulting peptides, larger than others, from 10 to 24 aa´, could be assembled by HLA class II molecules to be presented to CD4+ T cells (**Figure 1**) (Human Immuno-Peptidome Project, 2015; Mahdi, 2019; Purcell et al., 2019).

One of the concepts that antigen processing and subsequent presentation has a main effect on is the process named immune surveillance, which consist of the interaction of immune system with expressed intracellular and extracellular proteins. In this process, dendritic cells (DC), one of the most important APCs, are involved in scanning antigens on the surrounding tissues. After the antigen is recognized and internalized, the DCs are activated and migrate to the draining lymph nodes, where they can induce an adaptive immune response (Reis e Sousa, 2004). The antigen presentation pathway is completed when APCs process the internalized antigens and load their derived peptides onto MHC molecules (Embgenbroich and Burgdorf, 2018). Then, it is required to check endogenous expressed proteins (CD8 + T cells-HLA class I) or exogenous antigen presentation (CD8 + T cells-HLA class I for cross-presenting antigens and CD4 + T cells-HLA class II), due to constantly changes in the cell proteome that may trigger an immune response (Grabowska et al., 2018; Purcell et al., 2019).

Differences in the HLA subtypes of each class as well as differing antigen processing between the two classes lead to different peptides presented on these molecules. The nature of peptides also changes with the source of protein it is derived from. In recent years, HLA binding peptides have been analyzed to for databases (Altman et al., 1996; McHeyzer-Williams et al., 1996; Rodenko et al., 2006; Mommen et al., 2014; Bassani-Sternberg et al., 2015; Caron et al., 2015; Giam et al., 2015; Schittenhelm et al., 2015; Ternette et al., 2015; Bassani-Sternberg et al., 2016; Liepe et al., 2016; Mommen et al., 2016; Ternette et al., 2016; Abelin et al., 2017; Khodadoust et al., 2017; Shao et al., 2018), and the accuracy of the process as well as the role of antigen abundance, peptide length and post-translational modification have been investigated, to generate the complete immunopeptidome. Once peptides in the immunopeptidome is determined and characterized, these specific peptides can provide useful information for the development and design of several treatments like peptide-based vaccines as well as having a potential to be used as biomarkers in functional assays or enumeration of antigen-specific T cells (Altman et al., 1996; McHeyzer-Williams et al., 1996; Rodenko et al., 2006). Peptide-based vaccines are gaining attention in recent years, especially in the field of general vaccines against conserved regions of infectious pathogens (such as HIV, influenza and Plasmodium, among others) (Nardin et al., 2001; Parra-López et al., 2006; Purcell et al., 2007; Assarsson et al., 2008; Clemens et al., 2016; Sheikh et al., 2016). Also, this type of vaccines have a huge therapeutic potential in cancer with the discovery of many related novel epitopes, targeted by tumor-specific T cells after checkpoint suppression (Brennick et al., 2017; Verdegaal and van der Burg, 2017). For this goal, Human Immunopeptidome Project (HIPP) was been recently launched (https://www.hupo.org/Human-Immuno-Peptidome-Project), to provide a complete map of the human immunopeptidome and making the technology easy accessible, more robust and reproducible so that information will become available faster in translational clinical research (Vizcaíno et al., 2020).

In this mini-review, most relevant aspects about HLA-bound immunogenic peptides, the novel therapeutics approaches, the methodological strategies to identify MHC-I and II loaded peptides and the bioinformatic open-access databases in order to perform an *in silico* prediction of the potential target peptides with therapeutical interest it is detailed described.

## Immunopeptidomics: Concept, Applications, and Methodological Strategies

Immunopeptidomics is the large-scale study of peptides presented on HLA molecules. Thanks to the complete study of the endogenous peptides contained within a biological sample under defined conditions, the multitude of native peptides in a biological compartment can be exhaustively described with detailed features (ie. amino acid sequence, PTMs, peptide length, proteolysis processes) (Sirois et al., 2020).

In recent years, the study of immunopeptidomics has been of great interest to researchers in many scientific areas, from infectious and auto-immune diseases to oncology (Caron et al., 2017). In fact, Immunopeptidomics, based on mass spectrometry, currently is helping in the discovery of T cell targets against tumors, against autoimmune diseases and, more recently, against infectious pathogens for their application in the pandemic effects (ie. accelerate vaccine design and development, immune monitoring, engineering T cells (Caron et al., 2015).

Herein, the main features (in addition to advantages/disadvantages) of several currently developed approaches to decipher the peptides assembled in HLA molecules are critically discussed, as well as their applications in different biomedical research areas, noting that immunological knowledge and clinical translation might be highly similar regardless of the area of study, such as for cancer and infectious diseases.

### Infectious Diseases and Cancer Immunopeptidomics

Nowadays, it is well-known that many human health challenges are accompanied by disruptions in immune system and immune response. As the altered immune response is the key factor in the origin of many diseases (ie. auto-immune diseases, infectious diseases, chronic inflammation) and also innate and specific immune responses are involved in other pathologies with different ontogeny (ie. cancer, neurodegenerative,…); then, it is expected than multiple of methodological approaches for immunopeptidomics might be similar and commonly applied to identify antigen peptides in the pathological situations (Marko-Varga and LaBaer, 2017). Therefore, it seems that it is highly interesting to bring together an overview of complementary immunopeptidomics research in order to advance in the development of novel therapeutic approaches (ie. peptides vaccines,…) or to provide novel knowledge into the disease (Vance et al., 2017).

Given also the activation of immune response in infectious diseases is due to the presence of a pathogen; recently it is also been well-described that 20% of cancers could be caused by infectious agents, such as Helicobacter pylori, hepatitis C virus (HCV), Rouse Sarcoma Virus (RSV), Kaposi´s sarcoma-associated herpesvirus (KSHV), … as solid tumors and also in onco-hematological pathologies (such as chronic lymphocytic leukemia (CLL),…) (Mantovani et al., 2008; Kowalewski et al., 2015; Vance et al., 2017).

In addition, as the immune tolerance mechanism require a continuous steady-state between self- and non-self components, it is expected that the characterization of cell-cell communication, cellular microenviroment, cell migration, immune evasion and suppression, endothelial activation, inflammation initiation and evolution, phagocytosis, cell death mechanism, … are also critical on the immune tolerance (Vance et al., 2017). Thus, infectious disease studies influence cancer studies, and vice versa. Moreover, nowadays, due to the enormous success in cancer immunotherapy, it is becoming essential to understand the immunopeptidome because it is opening novel therapeutical opportunities to treat and prevent both, infectious and cancer diseases; among that infectious disease might be a risk factor to considered in the efficiency of multiple onco-immunotherapies (Acebes-Fernández et al., 2020).

In the last decade, many studies have been focused on the relationship between infectious diseases and cancers. One of these studies described the direct relationship between *Helicobacter pylori* infection and the cause of gastric cancer by evaluating the humoral immune response. In a previous study by L. Song et al. (2020), the humoral response to 1527 proteins (almost the entire immunoproteome of *Helicobacter pylori*) in 50 cases of gastric cancer was characterized, highlighting that decreased immune response to several proteins in gastric cancer which can reflect mucosal damage and low bacterial load. Among this, there is also evidence that 8% to 10% of gastric cancers are related to Epstein-Barr virus (EBV). Another study performed by L. Song et al. (2021), screened the humoral response to this virus in gastric cancer patients. As a consequence, of this screening, it is reported that Epstein-Barr virus-positive cancer can be detected by specific antibodies that can also be used for the diagnosis and treatment of the disease (Song et al., 2021).

Furthermore, the relation between cancer and infections has been also explored for the HLA-I and II molecules. In fact, several studies have found that protein fragments from bacteria invading tumor cells can be presented by HLA molecules on the surface of tumor cells and consequently these peptides are recognized by T-cells (Kalaora et al., 2021). Also immunopeptidomics showed that both antigen-presenting cells and tumor cells display bacterial and/or virus peptides on their cell surface by HLA molecules, which are specifically recognized by CD4+ and/or CD8+ T cells. Hence, these results could help in the selection of suitable bacterial or virus targets for cancer immunotherapy (Riemer, 2021). And more recently, in the last year, immunopeptidomics has also been successfully used to identify SARS-CoV-2 peptides; so then, the relationship between the binding capacity of viral peptides to 52 common MHC-I alleles and the mortality rate has been assessed, resulting in a high inverse relationship between peptides identified from the virus using a personal workflow called Ensemble-MHC (a consensus algorithm for the prediction of MHC-I peptides) and the mortality rate (Wilson et al., 2021).

Among these aspects, there is also a close relationship between onco-immunotherapies and infectious disease. Immune checkpoint inhibitors (ICI), one of the successful therapies in immunoncology, are under continuous investigation to be applied against T cell dysfunction in chronic viral infections (Barber et al., 2006). Similarly, CAR T-cell therapies being used in cancer therapy is also being repurposed for infectious diseases (Parida et al., 2015), mainly in design and develop CAR-T cells targeting pathogens infections. Since immunopeptidome could provide information about the peptides presented by the HLA system, which could be used to design tailored chimeric antigen receptors and vaccines that induce CD8+ T cells, in order to control infectious pathogens or fight tumors. An example of these approaches is the study of peptides presented by the human immunodeficiency virus type 1 (HIV-1) (Partridge et al., 2018) in which they found viral peptides specifically bound to HLA I and II molecules but did not elicit CD8+ T-cell responses. In a similar manner, in another immunopeptidomics and infection study, an attempt is being made to block the progression of pre-erythrocytic malaria, the asymptomatic stage of the disease, by means of a vaccine. So far, using live sporozoite-based vaccines is not feasible due to the great challenges. For this purpose, the identification of *Plasmodium falciparum* antigens expressed during this stage of the disease may be useful as vaccine candidates and improve the current state of treatment (Bettencourt, 2020).

Overall, immunopeptidome characterization, both for (and sometimes together) cancer and infectious diseases help to face challenges in vaccine development, overcome drawbacks and resistances to treatments, and help to increase the efficiency and efficacy of other already implemented onco-immunotherapies.

### Personalized Vaccines

The principle of producing personalized vaccines seems simple, but accurate and selective prediction of selective & specific disease peptide antigens for each patient remains as one of the major obstacles (Creech et al., 2018) It is estimated that each HLA heterodimer binds to thousands of peptides of allele-specific binding preference (Hunt et al., 1992; Rammensee et al., 1995; Rammensee et al., 1999; Bassani-Sternberg et al., 2015; Vita et al., 2015). Realizing the binding preference of each HLA heterodimer is the clue to successfully predict which antigens may cause specific T cell responses. For this reason, during the last decade, many efforts have been made in order to generate robust and reproducible methodological approaches for the specific identification of HLA loaded peptides which could be potential candidates for developing personalized peptide vaccines.

One of the promising therapeutic strategy in biomedicine are vaccines based on immunotherapy, especially active immunotherapy, which aims to activate the immune system *in vivo* and induce it to develop a high-specific response against exogenous and endogenous antigens. Following this way, therapeutic vaccines are divided in three types according to their content: cell vaccines, protein or peptide vaccines and genetic vaccines (made with DNA, RNA and viruses) (Acebes-Fernández et al., 2020). Here, the principal characteristics of each vaccine types will be revised from view of the role of HLA loaded peptides in the development of personalized peptide vaccines.

In cell vaccines, it is highlighted the Dendritic Cell (DC) vaccines, which are based on the intrinsic main features of DCs, commonly called professional APC. DCs, as professional antigen presenting cells, work in surrounding tissues where absorbs, process and present the pathogen and/or host antigenic peptides to primitive T lymphocytes in lymphatic organs through HLA. Therefore, although DC has a fundamental role in connecting innate and adaptive immunity, the functional characterization in DC determines that three signals are required to reach a complete and full activation. The first one is that for priming of T cells is necessary to proper loading MHC-peptide complexes. The second one, there must be an upregulation of costimulatory molecules (CD40, CD80, and CD86, for example). And the last one is the polarization of the immune response through the production of cytokines (Guo et al., 2013; Acebes-Fernández et al., 2020). There are numerous examples of these vaccines in difficult-to-treat diseases such as cancer, where DCs produced *in vitro* are used as tumoral vaccines. Mechanistically, human DC can be produced in culture from CD34+ hematopoietic progenitor cells or peripheral blood monocytes (Banchereau and Palucka, 2005). Thus, a DC vaccine is obtained by loading Tumor Associated Antigens (TAAs) onto the patient’s own DC, and then treating them with adjuvant. For example, Granulocyte-Macrophage Colony-Stimulating Factor (GM-CSF) is essential for *in vitro* production of monocyte-derived DC (Banchereau and Palucka, 2005). These cells require a maturation process, which is related to changes in the morphology and function of DCs. These procedures can improve the expression of MHC class I and II and co-stimulatory molecules, as well as increase the production of cytokines (Inaba et al., 1992). Then, these DCs are administered to patients to induce anti-tumor immunity. The first therapeutic cancer vaccine approved by the FDA was the DC vaccine Sipuleucel-T (Provenge™). It has successfully improved the survival rate of patients with a favorable toxicity profile in prostate cancer, opening a new paradigm for cancer treatment (Murphy et al., 1996; Small et al., 2006; Kantoff et al., 2010; Guo et al., 2013). Although there are more vaccines that have been used in clinical trials to treat other types of cancer such as melanoma, renal cell carcinoma and glioma (Nestle et al., 1998; HOLTL et al., 1999; Thurner et al., 1999; John et al., 2004; Small et al., 2006; Kantoff et al., 2010; Romano et al., 2011), further research is needed to prove its clinical efficacy and survival of patients with these types of cancers.

Another group of vaccines are the protein or peptide-based vaccines. Although initially these injections have been based on Tumor Associated Antigens (TAA), Cancer Germline Antigens (CGA), or Tumor Specific Antigens (TSA), together with some adjuvants, they could also be useful against infectious disease antigens. Protein or peptide-based vaccines include synthetic peptides with 20 to 30 amino acids from specific epitopes of tumor or infectious antigens. In these vaccines, the antigen could be adjusted to bind to immunogenic peptides, cytokines or antibodies (Pan et al., 2018). This type of vaccine is stable and not very expensive but has a major limitation which is the need to decipher the peptide epitopes to be used in these vaccines. Also immunosuppression present in the disease settings,as well as the weak immunogenicity of these antigens could be some disadvantages (Mocellin et al., 2009).

And the last type of vaccines is DNA vaccines: These are gene-based vaccines that use DNA (such as plasmids) or RNA (such as mRNA) (Mocellin et al., 2009). Viral DNA vectors can be used to deliver the cargo to the infiltrating somatic cells or DC (Guo et al., 2013). APC absorbs genetic material and translates them into cancer-specific antigens, thereby stimulating the immune system (Mocellin et al., 2009). Peptide or protein transcription and antigen presentation might be limited by the DNA/RNA delivery method for transfection efficiency and targeting (Mocellin et al., 2009). To administer the vaccines there are two methods: using viral vectors or by electroporation. Despite its effectiveness, it remains difficult to apply in routine clinical studies (Osada et al., 2012; Lee et al., 2015). Also it is necessary to report that the injection of live viruses can cause side effects and reduce the success of the vaccination due to clearance by antiviral antibodies in patients (Osada et al., 2012).

Considering the peptides and proteins used in cancer vaccines, in recent years, the NY-ESO-1 protein has been found to be a potential cancer vaccine antigen because of its high capacity to induce both humoral and cellular immune responses. In the study by Anna Pavlick et al. a phase I/II adjuvant clinical in resected high-risk melanomas was completed to improve the delivery of poly-ICLC as a constituent of the vaccine development. Poly-ICLC is a synthetic, stabilized, double-stranded RNA viral mimic capable of activating multiple innate immune receptors, activating CD4 and CD8 T cells and making it the optimal adjuvant for inducing *de novo* immune responses against tumor neoantigens (Pavlick et al., 2020).

The success of these vaccines depend on the right target selection and therefore, understanding peptide profiles presented on HLAs is essential for advancing the study of immunology, active immunotherapy, vaccine research and for further treatment development for any disease.

### Deciphering MHC-I and MHC-II Loaded Peptides

Recently, it has emerged of high clinical relevance to investigate how the HLA systems can affect susceptibility to infections, to immunotherapy response in oncology or in auto-immune diseases. For example, individual genetic variation will give different immune responses to a particular antigen, such as a microorganism, in a particular population. Bearing this in mind, the latest advances in this area has lead to specific identification of the peptides assembled in HLA molecules; for this purpose, several different strategies have been designed and developed which are briefly described in this review.

#### Systematic Isolation of HLA Molecules

**Figure 2:**
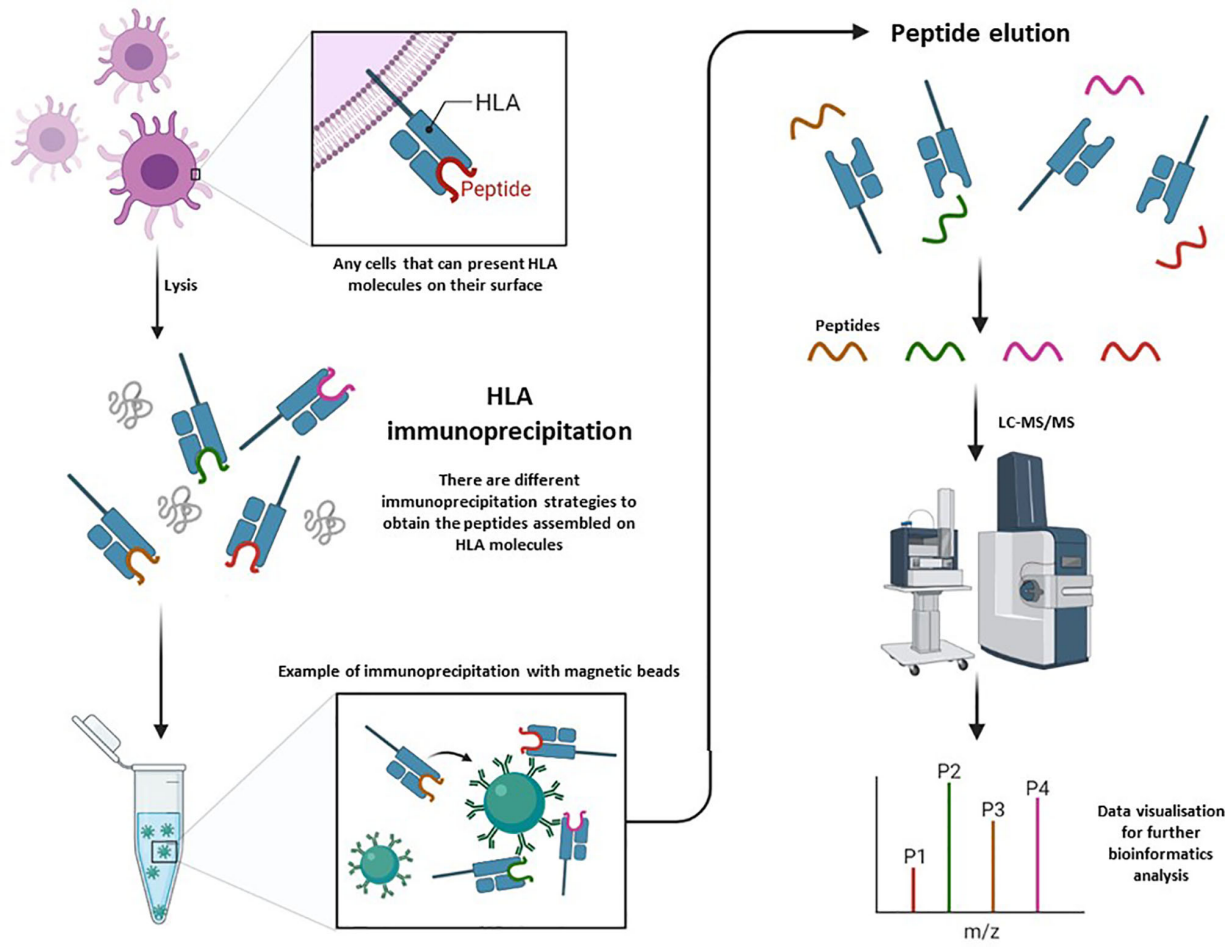
Global overview of the workflow for specific isolation of peptides from HLA molecules by immunochromatography and their further systematic characterization by LC-MS/MS.

By proteomics approaches, the identification of HLA loaded peptides require multiple sequential steps because of the relative low abundance of HLA molecules (except in APCs) and a low abundance of loaded peptides; among the particular features (such as size, fixed positions of hydrophobic/hydrophilic moieties, PTMs,…) of the loaded peptides derived from protein degradation (proteosome,…) and assembled in HLA groove…. Hence, it seems that the separation and enrichment of HLA molecules is critical, and the selective elution of loaded peptides is also a key point. Thus, several methodological strategies have been performed and here it is discussed a few of them; despite of it is still an area in continuous change, progress and evolution (**Figure 2**).

Most of the performed approaches are based on the specific and selective enrichment of HLA complexes by immuno-chromatography. Then, the HLA complex is immunopurified (IP) from the cell lysate (in presence of mild detergents), and further elution of peptides from the captured HLA complexes, which could be further analyzed, at high-resolution conditions, by liquid chromatography–mass spectrometry/mass spectrometry(LC-MS/MS). For this process, it is absolutely necessary to use optimal antibodies. Here, the most used are pan-HLA-I (Anti-Human HLA A, B, C: clone W6/32) and pan-HLA-II (Anti-Human HLA DR, DP, DQ: clone Tü39) (Bassani-Sternberg et al., 2010; Chong et al., 2018).

Regarding immunopurification, Chloe Chong et al. (2018) described a method based on sequential HLA purification by a chromatographic combination, starting by a pro-A beads (to capture endogenous antibodies), followed by an anti-HLA-I and/or anti-HLA-II antibodies coupled to pro-A beads to capture HLA class I and II, respectively. Then, HLA-I and HLA-II complexes are eluted and collected on a hydrophobic resin (C18) in order to selectively enrich loaded HLA peptides. The principal advantage of this strategy is that there are no intermediate steps, so it can continuously depleted endogenous antibodies and immunoaffinity purified class I and class II HLA complexes. Following this strategy, Chloe Chong et al. (2018) used B and T human cell lines and identified a total of 42,556 singular HLA class I peptides correlated to 8,975 proteins and 43,702 unrepeated HLA class II peptides from 4,501 proteins with a 1% false discovery rate (FDR). In both types of cell lines, the number of distinctive peptides changed from 3,293 to 13,696 for HLA I peptides and from 7,210 to 10,060 peptides for HLA II.

Another method is the direct immunoprecipitation using magnetic microspheres conjugated with anti-pan HLA I and II antibodies, respectively (Bassani-Sternberg et al., 2010; Chong et al., 2018). This procedure has the advantage of concentrating the cleft peptides of the HLA system thanks to the employment of super-paramagnetic microspheres conjugated to these highlighted antibodies. After separation of the peptides from the HLA molecules, the samples are eluted with an acid buffer and then the peptides are sequenced by mass spectrometry. If the whole exome or genome of the sample of interest is previously analysed to identify somatic mutations, then the results could be compared with the complete proteome (Chong et al., 2018; Kalaora and Samuels, 2019). In these studies, they compared several cancer cell lines with healthy ones, identified thousands of soluble HLA peptides, including some cancer specific peptides, shared among multiple several cell lines.

Recently, a novel procedure has been developed based on a mild acidic elution (MAE) of the HLA loaded peptides directly, in one single step, from the cells without any previous cell lysis and HLA selective enrichment. Here, thanks to the elution buffer thrown on the cells, the peptides carried by the cells of interest could be detected by further LC-MS/MS analysis. MAE strategy may be a cheap alternative to the other methods of immunoaffinity but is hindered by a large number of contaminating peptides not related with HLA molecules. By this strategy, Sturm T. et al., has identified the 50% common peptides between both approaches (MAE and immunoaffinity chromatography) as well as 22% of peptides identified only with MAE strategy (Sturm et al., 2020).

#### *In Silico* Prediction of HLA-I and HLA-II Loaded Peptides

**Figure 3:**
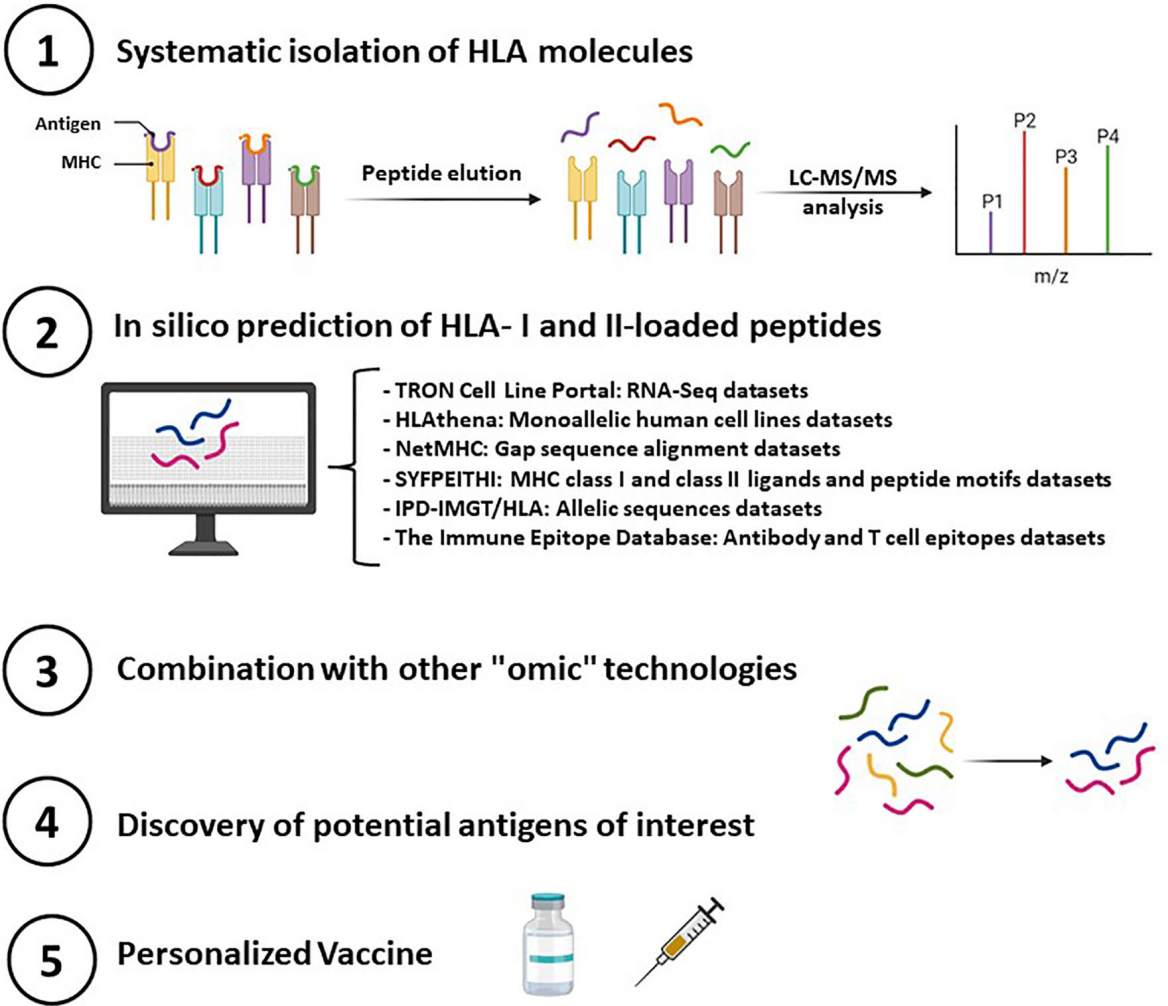
Essential steps in deciphering immunopeptides that elicit a T-cell response and therefore serve to design personalized peptide vaccines.

Having in mind that all human cells present HLA complexes and the critical role of HLA complexes in pathogen response and pathologies he precise and deep characterization of the immunopeptidome is highly important; hence, a synergically combination with multi-omics analysis (ie. metabolomics, genomics, proteomics, transcriptomics, epigenomics, among others) seems to be a powerful strategy for systematic determinations of immunopeptidomes. In addition to all of them, *in silico* prediction is a fundamental initial step to identify potential target neoantigens. For this purpose, it is crucial to carry out bioinformatic analysis that correlated the different databases and repositories of interest and integrate information from the immunopeptidome characterization (**Figure 3**).

Currently, several open-access bioinformatics tools are available for the prediction and selection of neoantigens by personalized proteogenomic workflows. One of them is called ProGeo-Neo (https://github.com/kbvstmd/ProGeo-neo) that allows neoantigen prediction and selection based on a customized proteogenomic pipeline (Li et al., 2020). ProGeo-Neo is based on the integration of three dataset packages: i.-RNA-seq data analysis, which could generate variant peptides; ii-HLA alleles are inferred from RNA-seq data; then it is possible to work only on selected HLA alleles; iii.- Neoantigen prediction is based on genomic and proteomics information which allows the screening of new antigens by LC-MS/MS and a neoantigen filtering through RNA expression and T cell receptor recognition (epitope). This novel pipeline is already being used as in Xiaoxiu Tan’s study, where a platform was developed to facilitate the screening and confirmation of potential neoantigens in cancer immunotherapy (Tan et al., 2020).

In a similar manner, several databases have been recently created for identified peptides assembled in HLA complexes; mainly based on the employed methodology (commonly LC-MS/MS) for the identification of the peptides. Herein, it is briefly described some relevant ones with relevance to oncology and infections (**Table 1** **)**.

**Table 1:** Main features related to current available databases focused on immunopeptidome characterization.

Database	Source Data	Info
TRON Cell Line Portal (http://celllines.tron-mainz.de/)	RNA-Seq datasets	Sample-details; Mutation-Data; Neo-Epitope-Data; Expression Data;
HLAthena (http://hlathena.tools/)	Monoallelic human cell lines datasets	Explore alleles; Select peptide length; Similarity based on allele motifs; Prediction peptides
NetMHC 4.1 (http://www.cbs.dtu.dk/services/NetMHC/)	Gap sequence alignment datasets	Alignment-based prediction algorithm; Peptide-MHC binding pattern; Length distribution of different HLA molecules
SYFPEITHI (http://www.syfpeithi.de/)	MHC class I and class II ligands and peptides motifs datasets	Ligand prediction; Correlates the prediction of T cell epitopes and HLA-loaded peptides
IPD-IMGT/HLA (https://www.ebi.ac.uk/ipd/imgt/hla/)	Allelic sequences datasets	Sequence alignment; Allele query; Sequence search tool; Cell query
The Immune Epitope Database (https://www.iedb.org/home_v3.php)	Antibody and T cell epitopes datasets	Prediction epitopes algorithm; Analysis epitopes tool

**TRON Cell Line Portal** or TCLP (http://celllines.tron-mainz.de/) (Scholtalbers et al., 2015) is a database that integrated the public RNA-Seq datasets of different exposed cell lines available in two repositories: The first set of data collected by Klijn et al. (2015) and the second one in The Cancer Cell Line Encyclopedia (CCLE). This database has been able to re-analyze accessible raw RNA-Seq datasets, determined the abundance and the type of HLA molecules as well as recognized virus and quantify the gene expression of 1,082 human cancer cell lines. Using all these available datasets of established HLA isotypes, cell lines- mutations and HLA prediction algorithms, Tron Cell Line Portal allows to predict the antigenic mutations in each analyzed human cell line. There are several studies in which they did typing of Human Leukocyte Antigens by High Throughput DNA and RNA Sequencing using TCLP, which includes an overview of approaches using high-throughput sequencing for HLA typing, as well as providing supplementary wet-lab protocols and *in silico* screening tools (Bukur, 2017; Boegel and Castle, 2019).

In this database, seq2HLA v2.2 is useful to identify the HLA isotypes (Boegel et al., 2014) which is able to calculate the four-digit HLA type from the RNA-Seq reads. It also generates two- and four-digit calls (Boegel et al., 2013; Boegel et al., 2014) with high precision. This public data includes HLA type data investigated by Adams et al. (2005), where sequence-based typing method (SBT) is used for HLA typing to determine the HLA class I and class II genotypes of the 60 cell lines used by National Cancer Institute (NCI-60). Using established HLA class I and II types in combination with systematic mutations will allow us to describe a register of possible neoepitopes candidates of HLA class I and II, respectively.

**HLAthena** (http://hlathena.tools/) This open-access portal is based on identification of HLA-I loaded peptides by exhaustive and systematic LC-MS/MS characterization. Here, more than 185,000 eluted peptides were analyzed from HLA A, B, C and G of 95 monoallelic human cell lines. The typical peptide motifs of each HLA allele was determined, as well as the unique and shared binding submotifs across alleles and the ones associated with a different peptide length. In addition, this database is developed by combining datasets with transcript abundance and the knowledge of peptide processing, which provides some prediction models of specific allele length for endogenous peptide presentation. These models predict HLA class I peptides compared with existing ligands tools and identify more than 75% of HLA I loaded peptides studied in 11 tumoral patients’ cells (melanoma, glioblastoma and clear cell renal cell carcinoma) with high accuracy. In summary, HLAthena database allows to systematically decipher the rules of presentation of endogenous antigens in tumoral cells (Abelin et al., 2017; Sarkizova et al., 2020). Other studies have used this database to predict and reduce the number of peptides to be studied in the face of the urgent need to develop a SARS-CoV-2 vaccine because the stimulation of an adequate immune response leading to protection is highly dependent on the presentation of epitopes to circulating T cells *via* the HLA complex. In this study 174 SARS-CoV-2 epitopes with high binding prediction scores were identified and validated to stably bind to 11 HLA allotypes (Prachar et al., 2020).

**NetMHC 4.1** (http://www.cbs.dtu.dk/services/NetMHC/) is a method for predicting peptides bound to MHC class I using gap sequence alignment. This method is based on artificial neural network to align the amino acid sequence of peptides assembled in the HLA complexes, which allows insertion and deletion in the alignment. Alignment-based prediction methods including deletions and insertions show higher throughput than strategies trained on single-length peptides (Buus et al., 2003; Nielsen et al., 2003; Andreatta and Nielsen, 2016). Similarly, they exemplify how the position of the deletion can help explain the peptide-MHC binding pattern, such as when a long peptide protrudes from the HLA groove or protrudes at either end. And they also demonstrated that this method can predict the length distribution of different HLA molecules, and used this prediction algorithm to quantify the reduction in the experimental workload required to identify potential epitopes. There are several studies that use this database to carry out studies of very diverse pathologies (Conley et al., 2018; Khanna and Rana, 2019). As an example, one study uses this database to predict T-cell epitopes of *Mycobacterium tuberculosis*. As the identification of T-cell or B-cell epitopes on the target antigen is the main goal in epitope-based vaccine design, immunological diagnostic tests and antibody development, it is essential to provide a robust and reproducible system that can assist in the diagnosis of *M. tuberculosis* (Khanna and Rana, 2019).

**SYFPEITHI** (http://www.syfpeithi.de/) This approach contains a collection of MHC class I and II ligands and peptide motifs from humans as well as additional species (such as apes, cows, chickens, and mice), which is constantly being updated. You can search for HLA alleles and motifs, and you can find natural ligands, T cell epitopes, source of proteins and organisms and their references. It includes links to The European Molecular Biology Laboratory (EMBL) and PubMed databases. In addition, ligand prediction can be used for various HLA allele products. The content of the database is limited to the availability of published datasets; however, it is highly useful to correlate the prediction of T cell epitopes and HLA-loaded peptides (Rammensee et al., 1999).

The prediction based on published motifs (such as natural ligands and cluster sequencing) considers the amino acids at anchor positions. Calculation of a reliable score is for anchoring is done by giving some amino acids of determined peptides specific values ​​depending on whether they are anchors, auxiliary anchors or preferred residues. Ideal anchoring is 10 points, unconventional anchoring is 6 to 8 points, auxiliary anchoring is 4 to 6, and residues are preferably 1 to 4 points. Also, some amino acids which have a molecular weight that is considered to have a negative outcome on binding capacity are between −1 and −3 (Rammensee et al., 1999). This novel database has already been used in the characterization (previously discussed) of HLA ligandome in chronic lymphocytic leukemia, among others (Kowalewski et al., 2015).

**IPD-IMGT/HLA** (https://www.ebi.ac.uk/ipd/imgt/hla/) includes 25,000 allelic sequences of more than 40 genes, which are encoded by the MHC of the human genome. The IPD-IMGT/HLA is a stable, highly accessible and easy-to-use database which provides access to many alternative sequences of this genetic system to the medical and scientific communities, essential for, for example, successful transplant results. The challenge for this database is to continue providing a highly selected sequence variation database while keeping an increased amount of submissions and the complexity of the sequence (Robinson et al., 2020).

This database has multiple tools, either its own or that have been incorporated from data libraries to the existing tools available from the European Bioinformatics Institute (https://www.ebi.ac.uk/) (EMBL-EBI) (Valentin et al., 2010). Among them, you can find: i.-Sequence alignment, ii.-Allele query, iii.-sequence search tool (FASTA and BLAST) and iv.-cell query (original materials) (Labarga et al., 2007; Valentin et al., 2010; Robinson et al., 2014; Albrecht et al., 2017; Madeira et al., 2019).

**The Immune Epitope Database (IEDB)** (https://www.iedb.org/home_v3.php) is an open-access database supported by the National Institute of Allergy and Infectious Diseases (NIAID). It catalogues, in the context of infectious disease, allergy, autoimmunity and transplantation, different experimental studies on antibody and T cell epitopes examined in humans as well as non-human primates and several animal species (Vita et al., 2019). The IEDB also hosts tools to help in the prediction and analysis of epitopes. In one recent study, they use the IEDB database to design a model for vaccine design by prediction of B-epitopes in perturbations in the sequence of a multitude of peptides, in various source or host organisms (González-Díaz et al., 2014).

#### Analysis of LC-MS/MS Data Sets of MHC-I and MHC-II Loaded Peptides

In addition to the information repositories about the peptides assembled in HLA molecules, tools for analyzing data extracted from LC-MS/MS characterization (including the different MS/MS instrumentation) and databases are increasingly common, including:

**DeepRescore** (https://github.com/bzhanglab/DeepRescore) is an immunopeptidomics data analysis device that supports deep learning-derived peptide characteristics to arrange peptide-spectrum matches (PSMs). It could take MS/MS raw data (in MGF format, for example) and recognize results from search engines as input. The last version supports four well-establish search engines like MS-GF+, Comet, X! Tandem and MaxQuant (Li et al., 2020). DeepRescore includes peptide characteristics derived from deep learning predictions (among which stand accurate retention time and MS/MS spectrum prediction) with previously used functions to reproduce peptide spectrum matching. Using two public immunopeptideomics data sets, it is demonstrated that compared with existing methods, the scoring performed by DeepRescore increases the accuracy and sensitivity of the recognition of MHC binding peptides and neoantigens. It also shows that, to a large extent, performance improvements are driven by features derived from deep learning. Thus, this post‐processing tool is freely available to the scientific community and can be used to identify sensitive and reproducible HLA binding peptides and neoantigens from immunopeptidomic datasets.

**PEAKS X PRO** (https://www.bioinfor.com/ Bioinformatics Solutions Inc., Waterloo, Ontario, Canada) is a commercially available software platform with several tools for the detection of peptide characteristics. Here, the three main characteristics are: DeepNovo is an extensive neural network model for *de novo* peptide sequencing. The DeepNovo design includes the latest developments in convolutional neural networks (CNN) and recurrent neural network (RNN) to learn the characteristics of peptides, as well as fragment ions and sequence patterns of tandem mass spectra. These systems are also integrated with local dynamic programming to solve the complex improvement tasks of *de novo* sequencing. DeepNovo has an accuracy growth at the amino acid level by 7.7% to 22.9% and an accuracy improvement at the peptide level by 38.1% to 64.0%. DeepNovo was used to automatically construct again the entire sequence of the mouse antibody light and heavy chain, without the need for others auxiliary databases to achieve 97.5% to 100% coverage and 97.2% to 99.5% accuracy. In addition, it can be retrained to modify any data source and supplies a whole end-to-end training and prediction solution for *de novo* sequencing problems (Tran et al., 2017). The second one is DeepIso, which combines the latest developments in CNN and RNN to estimate the peptide intensity and detect their features of different charge states. DeepIso consists of two different deep learning-based components, which can learn multiple levels of the high-dimensional data itself represented by multiple layers of neurons and can be adapted to new situations. The peptide characteristics list investigated with this model matches with 97.43% of high quality MS/MS identifications in a standard dataset (Zohora et al., 2019). The third one is DeepNovo-DIA, *de novo* peptide sequencing Independent data acquisition (DIA) method of mass spectrometry data. It also uses a neural network to capture cross m/z, retention time and intensity. Besides, DIA combined with peptide sequence pattern solves height problems multiple spectra, allowing us to identify novel peptides in human antigens and antibodies (Tran et al., 2019).

**MaxQuant** (https://www.maxquant.org/) is a quantitative proteomics software package developed for analyzing large mass-spectrometric data sets. It is the most used software with this type of raw data due to its versatility for handling and the interesting options with quantitative results. It is composed by a set of algorithms that extract information from raw MS data with high efficiency and sturdiness, capable of count elevated peptide identification rates, as well as high precision protein quantification for thousand proteins in complex proteomes (Cox and Mann, 2008). In recent years, the demand of MaxQuant has increased due to the great development of methodological strategies to identify peptides of interest in different pathologies (Schaab et al., 2012; Tyanova et al., 2015; Tyanova et al., 2016).

## Discussion

### Relevance of Immunopeptidomics Characterization in Oncology and Infections

For personalized medicine, a huge effort has been made to explore endogenous and exogenous processed ligands by diverse HLA heterodimers; which is an important from multiple points of view, such as: knowing the specific binding preferences of HLA could help predict secure and preferably immunogenic epitopes for independent patients with different pathologies. Technological and methodological advances (LC-MS/MS instrumentation, data search algorithms, acquisition methods, …) has increased the number and size of MS data repositories and their correlation with other multi-omics info (Vita et al., 2015; Fleri et al., 2017; Creech et al., 2018).

In addition, another advance has been the creation of international consortiums dedicated to establishing standardization strategies related to peptides isolated from HLA molecules. Hence, a few years ago, it was launched The Human Immuno‐Peptidome Project (HIPP) (https://www.hupo.org/Human-Immuno-Peptidome-Project), was launched under the umbrella of the Human Proteome Project Organization (HUPO), whose main goal is to use mass spectrometry technology to map the entire spectrum of peptides presented by HLA molecules and allow any immunologist, clinicians and other biomedical scientist to perform reliable analysis (Admon and Bassani-Sternberg, 2011; Caron et al., 2017). Within this framework, the immunogenicity of the predicted antigen will be confirmed through *in vitro* validation studies to evaluate the accuracy as well as the efficiency of present prediction algorithms investigated by numerous industrial and academic laboratories.

### Limitations of Immunopeptidomics Characterization

Bearing in mind all the methodological processes that must be carried out for the identification of immunogenic peptides assembled on HLA molecules, there are several limitations to be taken into account for their discovery.

The first limitation observed is the need to use an optimal lysis buffer, which allows us to obtain the maximum amount of membrane proteins, where the HLA molecules are subcellular located, and consequently the peptides assembled on them, to achieve a huge increment of relative protein abundance. If other specific lysis buffers are used for other subcellular compartments or for total protein extraction, maybe the relative abundance is not modified to enrich on HLA molecules with the subsequent decrease of peptides identification. In a similar manner, the elution buffer is also quite critical because it also might affect the yield on peptide isolation and identification (Chong et al., 2018).

Depending on the main goal of each immunopeptidome characterization, the immunoprecipitation strategy to be used must be taken into consideration; because another limitation observed is directly related to the pan-antibodies; because these pan-antibodies are optimal for maximizing the coverage, however, it also might lead to false HLA-restriction attribution to the eluted peptides (Bassani-Sternberg et al., 2010; Chong et al., 2018).

And finally, it must be taken into account that there are peptides with a weak and/or poor immunogenicity and involved in a possible immunosuppression, so that, only by using mass spectrometry, the identification of all the peptides bound to an HLA molecule may be limited (Purcell et al., 2019).

## Future Perspectives and Challenges

Despite the great advances in the knowledge and prediction of HLA-bound ligands, challenges remain because the antigens of interest currently represent a very small fraction of HLA-ligandome. Then, the integration of this knowledge with other multi-omics technologies is required, such as antigen identification based on whole exome sequencing and the subsequent predictive power of the algorithms that predict HLA ligand binding. However, these investigations emphasize the clinical possibilities of naturally processed HLA ligands based on MS sequencing, especially when merged with other “omics” strategies such as RNA-seq or next-generation sequencing (NGS) (Dutoit et al., 2012; Nelde et al., 2019).

Another challenge which needs to be overcome is the current need for plenty patient materials and the time frame required for patient-specific groups of HLA ligands and antigen analysis, which hinder and delay the implementation in the clinical environment and routines (Ott et al., 2017; Sahin et al., 2017; Creech et al., 2018). For example, tumor biopsy, HLA typing or whole exome sequencing and mutation detection may take about two weeks. At the same time, once the new antigen target is determined, HLA ligand enrichment, MS data collection and analysis may take several days to several weeks, while the design and manufacture of personalized vaccines may take several weeks. The total time limit for these procedures could be several months, which at this moment is incredibly lengthy for routine use in the clinic (Creech et al., 2018).

In addition, it is important to take into consideration that the response to personalized therapies will depend on the state of the immune system of each patient (Strønen et al., 2016). In order to understand how to optimize and adapt adaptive immune responses, and to improve the prediction and prioritization of antigenic determinants, further research is needed on the expected antigen T cell responses with TCR sequencing and epitope antigen prediction from TCR segments. Bearing this in mind, more studies which combine HLA-ligandome and TCR sequencing for a perfect recognition match between peptide loaded HLA and epitope mapping are required.

Predictions for potential antigens could provide candidates, in many recent studies predominantly for HLA class I epitopes due to the high accessibility of experimental data for class I prediction algorithms in contrast to class II. When CD4 + T cell responses have been studied in preclinical and clinical vaccination investigations (Dutoit et al., 2012; Kreiter et al., 2015; Sahin et al., 2017), it was shown that the processing and presentation of the HLA class II epitope may also play a critical role in the treatment of many diseases. However, although there are prediction algorithms for both classes, those of class II are less accurate because the peptide-binding groove allows longer peptides to bind, increasing the heterogeneity and complexity of epitope presentation (Depontieu et al., 2009; Mommen et al., 2016; Khodadoust et al., 2017). Therefore, more extensive analysis is required to better understand the characteristics of HLA class II bound peptides and the cellular procedures required for processing and presentation of class II epitopes (Kim et al., 2017).

Overall, improving prediction algorithms and combining MS HLA ligand profiles with other “omics” approaches is essential to create opportunities for customized peptide vaccines against various pathologies targeting antigens (self- and non-self) of interest and enable personalized immunotherapies (in oncology or infectious diseases) with large-scale clinical applications (Creech et al., 2018). For this reason, LC-MS/MS technology should continue to promote the improvement of epitope prediction and our knowledge of epitope processing and presentation for personalized immunotherapies which would transform the way patients with infectious, autoimmune diseases or even cancer are treated today.

## Author Contributions

Conceptualization: PJ-V, AL-V, VA-F, EM, RG, MF; Resources: PJ-V, APH, MLG-V, CA-H; Writing-original draft preparation: PJ-V, CA-H, HB, MF; Supervision: HB, EM, RG, MF; Funding acquisition: MF. All authors contributed to the article and approved the submitted version.

## Funding

We gratefully acknowledge financial support from the Spanish Health Institute Carlos III (ISCIII) for the grants: FIS PI17/01930 and CB16/12/00400. We also acknowledge Fondos FEDER (EU) and Junta Castilla-León (COVID19 grant COV20EDU/00187). Fundación Solórzano FS/38-2017. The Proteomics Unit belongs to ProteoRed, PRB3-ISCIII, supported by grant PT17/0019/0023, of the PE I + D + I 2017-2020, funded by ISCIII and FEDER. AL-V is supported by VIII Centenario-USAL PhD Program, PJ-V is supported by JCYL PhD Program and scholarship JCYL-EDU/601/2020.

## Conflict of Interest

The authors declare that the research was conducted in the absence of any commercial or financial relationships that could be construed as a potential conflict of interest.

## References

[B1] Abelin J. G. Keskin D. B. Sarkizova S. Hartigan C. R. Zhang W. Sidney J.. (2017). Mass Spectrometry Profiling of HLA-associated Peptidomes in Mono-Allelic Cells Enables More Accurate Epitope Prediction. Immunity 46 (2), 315–326. 10.1016/j.immuni.2017.02.007 28228285 PMC5405381

[B2] Abelin J. G. Keskin D. B. Sarkizova S. Hartigan C. R. Zhang W. Sidney J.. (2017). Mass Spectrometry Profiling of HLA-associated Peptidomes in Mono-Allelic Cells Enables More Accurate Epitope Prediction. Immunity 46 (2), 315–326. 10.1016/j.immuni.2017.02.007 28228285 PMC5405381

[B3] Acebes-Fernández V. Landeria-Viñuela A. Juanes-Velasco P. Hernández A. P. Otazo-Perez A. Manzano-Román R.. (2020). Nanomedicine and Onco-Immunotherapy: From the Bench to Bedside to Biomarkers. Nanomaterials 10 (7), 1274. 10.3390/nano10071274 PMC7407304 32610601

[B4] Adams S. Robbins F. M. Chen D. Wagage D. Holbeck S. L. Morse H. C.. (2005). HLA Class I and II Genotype of the NCI-60 Cell Lines. J. Trans. Med. 3 (1), 11. 10.1186/1479-5876-3-11 PMC555742 15748285

[B5] Admon A. Bassani-Sternberg M. (2011). The Human Immunopeptidome Project, a Suggestion for Yet Another Postgenome Next Big Thing. Mol. Cell. Proteomics 10 (10), O111–011833. 10.1074/mcp.O111.011833 PMC3205880 21813418

[B6] Albrecht V. Zweiniger C. Surendranath V. Lang K. Schöfl G. Dahl A.. (2017). Dual Redundant Sequencing Strategy: Full-length Gene Characterisation of 1056 Novel and Confirmatory HLA Alleles. Hla 90 (2), 79–87. 10.1111/tan.13057 28547825 PMC6084308

[B7] Altman J. D. Moss P. A. Goulder P. J. Barouch D. H. McHeyzer-Williams M. G. Bell J. I.. (1996). Phenotypic Analysis of Antigen-Specific T Lymphocytes. Science 274 (5284), 94–96. 10.1007/BF02246016 8810254

[B8] Andreatta M. Nielsen M. (2016). Gapped Sequence Alignment Using Artificial Neural Networks: Application to the MHC Class I System. Bioinformatics 32 (4), 511–517. 10.1093/bioinformatics/btv639 26515819 PMC6402319

[B9] Assarsson E. Bui H. H. Sidney J. Zhang Q. Glenn J. Oseroff C.. (2008). Immunomic Analysis of the Repertoire of T-cell Specificities for Influenza A Virus in Humans. J. Virol. 82 (24), 12241–12251. 10.1128/JVI.01563-08 18842709 PMC2593359

[B10] Banchereau J. Palucka A. K. (2005). Dendritic Cells as Therapeutic Vaccines Against Cancer. Nat. Rev. Immunol. 5 (4), 296–306. 10.1038/nri1592 15803149

[B11] Barber D. L. Wherry E. J. Masopust D. Zhu B. Allison J. P. Sharpe A. H.. (2006). Restoring Function in Exhausted CD8 T Cells During Chronic Viral Infection. Nature 439 (7077), 682–687. 10.1038/nature04444 16382236

[B12] Bassani-Sternberg M. Barnea E. Beer I. Avivi I. Katz T. Admon A. (2010). Soluble Plasma HLA Peptidome as a Potential Source for Cancer Biomarkers. Proc. Natl. Acad. Sci. 107 (44), 18769–18776. 10.1073/pnas.1008501107 20974924 PMC2973870

[B13] Bassani-Sternberg M. Bräunlein E. Klar R. Engleitner T. Sinitcyn P. Audehm S.. (2016). Direct Identification of Clinically Relevant Neoepitopes Presented on Native Human Melanoma Tissue by Mass Spectrometry. Nat. Commun. 7 (1), 1–16. 10.1038/ncomms13404 PMC5121339 27869121

[B14] Bassani-Sternberg M. Pletscher-Frankild S. Jensen L. J. Mann M. (2015). Mass Spectrometry of Human Leukocyte Antigen Class I Peptidomes Reveals Strong Effects of Protein Abundance and Turnover on Antigen Presentation. Mol. Cell. Proteomics 14 (3), 658–673. 10.1074/mcp.M114.042812 25576301 PMC4349985

[B15] Bassani-Sternberg M. Pletscher-Frankild S. Jensen L. J. Mann M. (2015). Mass Spectrometry of Human Leukocyte Antigen Class I Peptidomes Reveals Strong Effects of Protein Abundance and Turnover on Antigen Presentation. Mol. Cell. Proteomics 14 (3), 658–673. 10.1074/mcp.M114.042812 25576301 PMC4349985

[B16] Bettencourt P. (2020). Current Challenges in the Identification of Pre-Erythrocytic Malaria Vaccine Candidate Antigens. Front. Immunol. 11, 190. 10.3389/fimmu.2020.00190 32153565 PMC7046804

[B17] Boegel S. Castle J. C. (2019). Human Leukocyte Antigen Typing Using High-Throughput DNA and RNA Sequencing and Application for Cell Line Identification. Adv. Mol. Pathol. 2 (1), 187–199. 10.1016/j.yamp.2019.07.013

[B18] Boegel S. Löwer M. Bukur T. Sahin U. Castle J. C. (2014). A Catalog of HLA Type, HLA Expression, and Neo-Epitope Candidates in Human Cancer Cell Lines. Oncoimmunology 3 (8), e954893. 10.4161/21624011.2014.954893 25960936 PMC4355981

[B19] Boegel S. Löwer M. Schäfer M. Bukur T. De Graaf J. Boisguérin V.. (2013). HLA Typing From RNA-Seq Sequence Reads. Genome Med. 4 (12), 102. 10.1186/gm4039 PMC4064318 23259685

[B20] Brennick C. A. George M. M. Corwin W. L. Srivastava P. K. Ebrahimi-Nik H. (2017). Neoepitopes as Cancer Immunotherapy Targets: Key Challenges and Opportunities. Immunotherapy 9 (4), 361–371. 10.2217/imt-2016-0146 28303769

[B21] Bukur T. (2017). Rna-Seq Based Decomposition of Human Cell Lines and Primary Tumors for the Identification and Quantification of Viral Expression (Doctoral Dissertation). Faculty of Biology and the Medical Center of the Johannes Gutenberg-University Mainz, and at TRON – Translational Oncology at the University Medical Center of the Johannes Gutenberg-University Mainz gGmbH

[B22] Buus S. Lauemøller S. L. Worning P. Kesmir C. Frimurer T. Corbet S.. (2003). Sensitive Quantitative Predictions of peptide-MHC Binding by a ‘Query by Committee’artificial Neural Network Approach. Tissue Antigens 62 (5), 378–384. 10.1034/j.1399-0039.2003.00112.x 14617044

[B23] Caron E. Aebersold R. Banaei-Esfahani A. Chong C. (2017). A Case for a Human Immuno-Peptidome Project Consortium. Immunity 47, 203 208. 10.1016/j.immuni.2017.07.010 28813649

[B24] Caron E. Espona L. Kowalewski D. J. Schuster H. Ternette N. Alpizar A.. (2015). An Open-Source Computational and Data Resource to Analyze Digital Maps of Immunopeptidomes. Elife 4, e07661. 10.7554/eLife.07661.001 PMC4507788 26154972

[B25] Caron E. Kowalewski D. J. Koh C. C. Sturm T. Schuster H. (2015). Analysis of Major Histocompatibility Complex (Mhc) Immunopeptidomes Using Mass Spectrometry. Mol. Cell Proteomics 14, 3105–3117. 10.1074/mcp.O115.052431 26628741 PMC4762616

[B26] Chong C. Marino F. Pak H. Racle J. Daniel R. T. Müller M.. (2018). High-throughput and Sensitive Immunopeptidomics Platform Reveals Profound Interferonγ-Mediated Remodeling of the Human Leukocyte Antigen (HLA) Ligandome. Mol. Cell. Proteomics 17 (3), 533–548. 10.1074/mcp.TIR117.000383 29242379 PMC5836376

[B27] Clemens E. B. Grant E. J. Wang Z. Gras S. Tipping P. Rossjohn J.. (2016). Towards Identification of Immune and Genetic Correlates of Severe Influenza Disease in Indigenous Australians. Immunol. Cell Biol. 94 (4), 367–377. 10.1038/icb.2015.93 26493179 PMC4840236

[B28] Conley A. P. Van Anh Trinh C. M. Z. Posey K. Martinez J. D. Arrieta O. G. Wang W. L.. (2018). Positive Tumor Response to Combined Checkpoint Inhibitors in a Patient With Refractory Alveolar Soft Part Sarcoma: A Case Report. J. Global Oncol. 4. 10.1200/JGO.2017.009993 PMC6180844 30241159

[B29] Cox J. Mann M. (2008). MaxQuant Enables High Peptide Identification Rates, Individualized Ppb-Range Mass Accuracies and Proteome-Wide Protein Quantification. Nat. Biotechnol. 26 (12), 1367–1372. 10.1038/nbt.1511 19029910

[B30] Creech A. L. Ting Y. S. Goulding S. P. Sauld J. F. Barthelme D. Rooney M. S.. (2018). The Role of Mass Spectrometry and Proteogenomics in the Advancement of HLA Epitope Prediction. Proteomics 18 (12), 1700259. 10.1002/pmic.201700259 PMC6033110 29314742

[B31] Depontieu F. R. Qian J. Zarling A. L. McMiller T. L. Salay T. M. Norris A.. (2009). Identification of Tumor-Associated, MHC Class II-restricted Phosphopeptides as Targets for Immunotherapy. Proc. Natl. Acad. Sci. 106 (29), 12073–12078. 10.1073/pnas.0903852106 19581576 PMC2715484

[B32] Dutoit V. Herold-Mende C. Hilf N. Schoor O. Beckhove P. Bucher J.. (2012). Exploiting the Glioblastoma Peptidome to Discover Novel Tumour-Associated Antigens for Immunotherapy. Brain 135 (4), 1042–1054. 10.1093/brain/aws042 22418738

[B33] Embgenbroich M. Burgdorf S. (2018). Current Concepts of Antigen Cross-Presentation. Front. Immunol. 9, 1643. 10.3389/fimmu.2018.01643 30061897 PMC6054923

[B34] Fleri W. Paul S. Dhanda S. K. Mahajan S. Xu X. Peters B.. (2017). The Immune Epitope Database and Analysis Resource in Epitope Discovery and Synthetic Vaccine Design. Front. Immunol. 8, 278. 10.3389/fimmu.2017.00278 28352270 PMC5348633

[B35] Giam K. Ayala-Perez R. Illing P. T. Schittenhelm R. B. Croft N. P. Purcell A. W.. (2015). A Comprehensive Analysis of Peptides Presented by HLA-A1. Tissue Antigens 85 (6), 492–496. 10.1111/tan.12565 25880248

[B36] González-Díaz H. Pérez-Montoto L. G. Ubeira F. M. (2014). Model for Vaccine Design by Prediction of B-epitopes of IEDB Given Perturbations in Peptide Sequence, *In Vivo* Process, Experimental Techniques, and Source or Host Organisms. J. Immunol. Res. 2014. 10.1155/2014/768515 PMC3987976 24741624

[B37] Grabowska J. Lopez-Venegas M. A. Affandi A. J. Den Haan J. M. (2018). CD169+ Macrophages Capture and Dendritic Cells Instruct: The Interplay of the Gatekeeper and the General of the Immune System. Front. Immunol. 9, 2472. 10.3389/fimmu.2018.02472 30416504 PMC6212557

[B38] Guo C. Manjili M. H. Subjeck J. R. Sarkar D. Fisher P. B. Wang X. Y. (2013). “Therapeutic Cancer Vaccines: Past, Present, and Future,” in Advances in Cancer Research, vol. 119. (Academic Press), 421–475. 23870514 10.1016/B978-0-12-407190-2.00007-1 PMC3721379

[B39] HOLTL L. RIESER C. PAPESH C. RAMONER R. HEROLD M. KLOCKER H.. (1999). Cellular and Humoral Immune Responses in Patients With Metastatic Renal Cell Carcinoma After Vaccination With Antigen Pulsed Dendritic Cells. J. Urol. 161 (3), 777–782. 10.1016/S0022-5347(01)61767-1 10022683

[B40] Human Immuno-Peptidome Project (2015). Available at: https://www.hupo.org/Human-Immuno-Peptidome-Project.

[B41] Hunt D. F. Henderson R. A. Shabanowitz J. Sakaguchi K. Michel H. Sevilir N.. (1992). Characterization of Peptides Bound to the Class I MHC Molecule HLA-A2. 1 by mass spectrometry. Science 255 (5049), 1261–1263. 10.1126/science.1546328 1546328

[B42] Inaba K. Inaba M. Romani N. Aya H. Deguchi M. Ikehara S.. (1992). Generation of Large Numbers of Dendritic Cells From Mouse Bone Marrow Cultures Supplemented With Granulocyte/Macrophage Colony-Stimulating Factor. J. Exp. Med. 176 (6), 1693–1702. 10.1084/jem.176.6.1693 1460426 PMC2119469

[B43] John S. Y. Liu G. Ying H. Yong W. H. Black K. L. Wheeler C. J. (2004). Vaccination With Tumor Lysate-Pulsed Dendritic Cells Elicits Antigen-Specific, Cytotoxic T-cells in Patients With Malignant Glioma. Cancer Res. 64 (14), 4973–4979. 10.1158/0008-5472.CAN-03-3505 15256471

[B44] Kalaora S. Nagler A. Nejman D. Alon M. Barbolin C. Barnea E.. (2021). Identification of Bacteria-Derived HLA-bound Peptides in Melanoma. Nature 592 (7852), 1–6. 10.1038/s41579-021-00551-6 PMC9717498 33731925

[B45] Kalaora S. Samuels Y. (2019). “Cancer Exome-Based Identification of Tumor Neo-Antigens Using Mass Spectrometry,” in Cancer Immunosurveillance (New York, NY: Humana Press), 203–214. 10.1007/978-1-4939-8885-3_14 30465205

[B46] Kantoff P. W. Higano C. S. Shore N. D. Berger E. R. Small E. J. Penson D. F.. (2010). Sipuleucel-T Immunotherapy for Castration-Resistant Prostate Cancer. New Engl. J. Med. 363 (5), 411–422. 10.1056/NEJMoa1001294 20818862

[B47] Khanna D. Rana P. S. (2019). Ensemble Technique for Prediction of T-cell Mycobacterium Tuberculosis Epitopes. Interdiscip. Sci.: Comput. Life Sci. 11 (4), 611–627. 10.1007/s12539-018-0309-0 30406342

[B48] Khodadoust M. S. Olsson N. Wagar L. E. Haabeth O. A. Chen B. Swaminathan K.. (2017). Antigen Presentation Profiling Reveals Recognition of Lymphoma Immunoglobulin Neoantigens. Nature 543 (7647), 723–727. 10.1038/nature21433 28329770 PMC5808925

[B49] Kim A. Boronina T. N. Cole R. N. Darrah E. Sadegh-Nasseri S. (2017). Distorted Immunodominance by Linker Sequences or Other Epitopes From a Second Protein Antigen During Antigen-Processing. Sci. Rep. 7, 46418. 10.1038/srep46418 28422163 PMC5396073

[B50] Klijn C. Durinck S. Stawiski E. W. Haverty P. M. Jiang Z. Liu H.. (2015). A Comprehensive Transcriptional Portrait of Human Cancer Cell Lines. Nat. Biotechnol. 33 (3), 306–312. 10.1038/nbt.3080 25485619

[B51] Kowalewski D. J. Schuster H. Backert L. Berlin C. Kahn S. Kanz L.. (2015). HLA Ligandome Analysis Identifies the Underlying Specificities of Spontaneous Antileukemia Immune Responses in Chronic Lymphocytic Leukemia (CLL). Proc. Natl. Acad. Sci. 112 (2), E166–E175. 10.1073/pnas.1416389112 25548167 PMC4299203

[B52] Kreiter S. Vormehr M. Van de Roemer N. Diken M. Löwer M. Diekmann J.. (2015). Mutant MHC Class II Epitopes Drive Therapeutic Immune Responses to Cancer. Nature 520 (7549), 692–696. 10.1038/nature14426 25901682 PMC4838069

[B53] Labarga A. Valentin F. Anderson M. Lopez R. (2007). Web Services at the European Bioinformatics Institute. Nucleic Acids Res. 35 (suppl_2), W6–W11. 10.1093/nar/gkm291 17576686 PMC1933145

[B54] Lee S. H. Danishmalik S. N. Sin J. I. (2015). DNA Vaccines, Electroporation and Their Applications in Cancer Treatment. Hum. Vaccines Immunother. 11 (8), 1889–1900. 10.1080/21645515.2015.1035502 PMC4635908 25984993

[B55] Liepe J. Marino F. Sidney J. Jeko A. Bunting D. E. Sette A.. (2016). A Large Fraction of HLA Class I Ligands are Proteasome-Generated Spliced Peptides. Science 354 (6310), 354–358. 10.1126/science.aaf4384 27846572

[B56] Li K. Jain A. Malovannaya A. Wen B. Zhang B. (2020). Deeprescore: Leveraging Deep Learning to Improve Peptide Identification in Immunopeptidomics. Proteomics 20 (21-22), 1900334. 10.1002/pmic.201900334 PMC7718998 32864883

[B57] Li Y. Wang G. Tan X. Ouyang J. Zhang M. Song X.. (2020). ProGeo-neo: A Customized Proteogenomic Workflow for Neoantigen Prediction and Selection. BMC Med. Genomics 13 (5), 1–11. 10.1186/s12920-020-0683-4 32241270 PMC7118832

[B58] Madeira F. Park Y. M. Lee J. Buso N. Gur T. Madhusoodanan N.. (2019). The EMBL-EBI Search and Sequence Analysis Tools APIs in 2019. Nucleic Acids Res. 47 (W1), W636–W641. 10.1093/nar/gkz268 30976793 PMC6602479

[B59] Mahdi B. M. (2019). “Introductory Chapter: Concept of Human Leukocyte Antigen (Hla),” in Human Leukocyte Antigen (HLA). Intechopen. 1–8.

[B60] Mantovani A. Allavena P. Sica A. Balkwill F. (2008). Cancer-Related Inflammation. nature 454 (7203), 436–444. 10.1038/nature07205 18650914

[B61] Marko-Varga G. LaBaer J. (2017). The Immune System and the Proteome. J. Proteome Res. 16, 1a. 10.1021/acs.jproteome.6b00607 28056507

[B62] McHeyzer-Williams M. G. Altman J. D. Davis M. M. (1996). Enumeration and Characterization of Memory Cells in the TH Compartment. Immunol. Rev. 150, 5. 10.1111/j.1600-065X.1996.tb00693.x 8782699

[B63] Mocellin S. Pilati P. Nitti D. (2009). Peptide-Based Anticancer Vaccines: Recent Advances and Future Perspectives. Curr. Med. Chem. 16 (36), 4779–4796. 10.2174/092986709789909648 19929787

[B64] Mommen G. P. Frese C. K. Meiring H. D. van Gaans-van den Brink J. de Jong A. P. van Els C. A.. (2014). Expanding the Detectable HLA Peptide Repertoire Using Electron-Transfer/Higher-Energy Collision Dissociation (Ethcd). Proc. Natl. Acad. Sci. 111 (12), 4507–4512. 10.1073/pnas.1321458111 24616531 PMC3970485

[B65] Mommen G. P. Marino F. Meiring H. D. Poelen M. C. Mohammed S. Heck A. J.. (2016). Sampling From the Proteome to the Human Leukocyte antigen-DR (Hla-DR) Ligandome Proceeds *Via* High Specificity. Mol. Cell. Proteomics 15 (4), 1412–1423. 10.1074/mcp.M115.055780 26764012 PMC4824864

[B66] Murphy G. Tjoa B. Ragde H. Kenny G. Boynton A. (1996). Phase I Clinical Trial: T-cell Therapy for Prostate Cancer Using Autologous Dendritic Cells Pulsed With HLA-A0201-specific Peptides From Prostate-Specific Membrane Antigen. Prostate 29 (6), 371–380. 10.1002/(SICI)1097-0045(199612)29:6<371::AID-PROS5>3.0.CO;2-B 8977634

[B67] Nardin E. H. Calvo-Calle J. M. Oliveira G. A. Nussenzweig R. S. Schneider M. Tiercy J. M.. (2001). A Totally Synthetic Polyoxime Malaria Vaccine Containing Plasmodium Falciparum B Cell and Universal T Cell Epitopes Elicits Immune Responses in Volunteers of Diverse HLA Types. J. Immunol. 166 (1), 481–489. 10.4049/jimmunol.166.1.481 11123327

[B68] Nelde A. Kowalewski D. J. Stevanović S. (2019). “Purification and Identification of Naturally Presented MHC Class I and II Ligands,” in Antigen Processing (New York, NY: Humana), 123–136. 10.1007/978-1-4939-9450-2_10 31147937

[B69] Nestle F. O. Alijagic S. Gilliet M. Sun Y. Grabbe S. Dummer R.. (1998). Vaccination of Melanoma Patients With Peptide-or Tumorlysate-Pulsed Dendritic Cells. Nat. Med. 4 (3), 328–332. 10.1038/nm0398-328 9500607

[B70] Nielsen M. Lundegaard C. Worning P. Lauemøller S. L. Lamberth K. Buus S.. (2003). Reliable Prediction of T-cell Epitopes Using Neural Networks With Novel Sequence Representations. Protein Sci. 12 (5), 1007–1017. 10.1110/ps.0239403 12717023 PMC2323871

[B71] Osada T. Morse M. A. Hobeika A. Lyerly H. K. (2012). “Novel Recombinant Alphaviral and Adenoviral Vectors for Cancer Immunotherapy,” in Seminars in Oncology, vol. 39. (WB Saunders), 305–310. 22595053 10.1053/j.seminoncol.2012.02.013 PMC3607360

[B72] Ott P. A. Hu Z. Keskin D. B. Shukla S. A. Sun J. Bozym D. J.. (2017). An Immunogenic Personal Neoantigen Vaccine for Patients With Melanoma. Nature 547 (7662), 217–221. 10.1038/nature22991 28678778 PMC5577644

[B73] Pan R. Y. Chung W. H. Chu M. T. Chen S. J. Chen H. C. Zheng L.. (2018). Recent Development and Clinical Application of Cancer Vaccine: Targeting Neoantigens. J. Immunol. Res. 2018. 10.1155/2018/4325874 PMC6313977 30662919

[B74] Parida S. K. Poiret T. Zhenjiang L. Meng Q. Heyckendorf J. Lange C.. (2015). T-Cell Therapy: Options for Infectious Diseases. Clin. Infect. Dis. 61 (suppl_3), S217–S224. 10.1093/cid/civ615 PMC4583575 26409284

[B75] Parra-López C. Calvo-Calle J. M. Cameron T. O. Vargas L. E. Salazar L. M. Patarroyo M. E.. (2006). Major Histocompatibility Complex and T Cell Interactions of a Universal T Cell Epitope From Plasmodium Falciparum Circumsporozoite Protein. J. Biol. Chem. 281 (21), 14907–14917. 10.1074/jbc.M511571200 16565072

[B76] Partridge T. Nicastri A. Kliszczak A. E. Yindom L. M. Kessler B. M. Ternette N.. (2018). Discrimination Between Human Leukocyte Antigen Class I-bound and Co-Purified HIV-derived Peptides in Immunopeptidomics Workflows. Front. Immunol. 9, 912. 10.3389/fimmu.2018.00912 29780384 PMC5946011

[B77] Pavlick A. Blazquez A. B. Meseck M. Lattanzi M. Ott P. A. Marron T. U.. (2020). Combined Vaccination With NY-ESO-1 Protein, poly-ICLC, and Montanide Improves Humoral and Cellular Immune Responses in High-Risk Melanoma Patients. Cancer Immunol. Res. 8 (1), 70. 10.1158/2326-6066.CIR-19-0545 31699709 PMC6946846

[B78] Prachar M. Justesen S. Steen-Jensen D. B. Thorgrimsen S. P. Jurgons E. Winther O.. (2020). Covid-19 Vaccine Candidates: Prediction and Validation of 174 SARS-CoV-2 Epitopes. bioRxiv. 10.1101/2020.03.20.000794 PMC7686376 33235258

[B79] Purcell A. W. McCluskey J. Rossjohn J. (2007). More Than One Reason to Rethink the Use of Peptides in Vaccine Design. Nat. Rev. Drug Discovery 6 (5), 404–414. 10.1038/nrd2224 17473845

[B80] Purcell A. W. Ramarathinam S. H. Ternette N. (2019). Mass Spectrometry–Based Identification of MHC-bound Peptides for Immunopeptidomics. Nat. Protoc. 14 (6), 1687. 10.1038/s41596-019-0133-y 31092913

[B81] Rammensee H. G. Bachmann J. Emmerich N. P. N. Bachor O. A. Stevanović S. S. Y. F. P. E. I. T. H. I. (1999). SYFPEITHI: Database for MHC Ligands and Peptide Motifs. Immunogenetics 50 (3-4), 213–219. 10.1007/s002510050595 10602881

[B82] Rammensee H. G. Bachmann J. Emmerich N. P. N. Bachor O. A. Stevanović S. S. Y. F. P. E. I. T. H. I. (1999). SYFPEITHI: Database for MHC Ligands and Peptide Motifs. Immunogenetics 50 (3-4), 213–219. 10.1007/s002510050595 10602881

[B83] Rammensee H. G. Friede T. Stevanović S. (1995). MHC Ligands and Peptide Motifs: First Listing. Immunogenetics 41 (4), 178–228. 10.1007/BF00172063 7890324

[B84] Reis e Sousa C. (2004). “Toll-Like Receptors and Dendritic Cells: for Whom the Bug Tolls,” in Seminars in Immunology, vol. 16. (Academic Press), 27–34. 14751761 10.1016/j.smim.2003.10.004

[B85] Riemer A. B. (2021). Bacterial Peptides Presented on Tumour Cells Could be Immunotherapy Targets. Nature 592, 28–29. 10.1038/d41586-021-00640-9 33731901

[B86] Robinson J. Barker D. J. Georgiou X. Cooper M. A. Flicek P. Marsh S. G. (2020). Ipd-IMGT/HLA Database. Nucleic Acids Res. 48 (D1), D948–D955. 10.1093/nar/gkz950 31667505 PMC7145640

[B87] Robinson J. Halliwell J. A. Marsh S. G. (2014). “IMGT/HLA and the Immuno Polymorphism Database,” in Immunoinformatics (New York, NY: Humana Press), 109–121.

[B88] Rodenko B. Toebes M. Hadrup S. R. Van Esch W. J. Molenaar A. M. Schumacher T. N.. (2006). Generation of Peptide–MHC Class I Complexes Through UV-mediated Ligand Exchange. Nat. Protoc. 1 (3), 1120. 10.1038/nprot.2006.121 17406393

[B89] Romano E. Rossi M. Ratzinger G. de Cos M. A. Chung D. J. Panageas K. S.. (2011). Peptide-Loaded Langerhans Cells, Despite Increased IL15 Secretion and T-cell Activation *In Vitro*, Elicit Antitumor T-cell Responses Comparable to Peptide-Loaded Monocyte-Derived Dendritic Cells *In Vivo* . Clin. Cancer Res. 17 (7), 1984–1997. 10.1158/1078-0432.CCR-10-3421 21355077 PMC3743659

[B90] Sahin U. Derhovanessian E. Miller M. Kloke B. P. Simon P. Löwer M.. (2017). Personalized RNA Mutanome Vaccines Mobilize Poly-Specific Therapeutic Immunity Against Cancer. Nature 547 (7662), 222–226. 10.1038/nature23003 28678784

[B91] Sarkizova S. Klaeger S. Le P. M. Li L. W. Oliveira G. Keshishian H.. (2020). A Large Peptidome Dataset Improves HLA Class I Epitope Prediction Across Most of the Human Population. Nat. Biotechnol. 38 (2), 199–209. 10.1038/s41587-019-0322-9 31844290 PMC7008090

[B92] Schaab C. Geiger T. Stoehr G. Cox J. Mann M. (2012). Analysis of High Accuracy, Quantitative Proteomics Data in the MaxQB Database. Mol. Cell. Proteomics 11 (3), M111–014068. 10.1074/mcp.M111.014068 PMC3316731 22301388

[B93] Schittenhelm R. B. Sian T. C. L. K. Wilmann P. G. Dudek N. L. Purcell A. W. (2015). Revisiting the Arthritogenic Peptide Theory: Quantitative Not Qualitative Changes in the Peptide Repertoire of HLA–B27 Allotypes. Arthritis Rheumatol. 67 (3), 702–713. 10.1002/art.38963 25418920

[B94] Scholtalbers J. Boegel S. Bukur T. Byl M. Goerges S. Sorn P.. (2015). TCLP: An Online Cancer Cell Line Catalogue Integrating HLA Type, Predicted Neo-Epitopes, Virus and Gene Expression. Genome Med. 7 (1), 1–7. 10.1186/s13073-015-0240-5 26589293 PMC4653878

[B95] Shao W. Pedrioli P. G. Wolski W. Scurtescu C. Schmid E. Vizcaíno J. A.. (2018). The SysteMHC Atlas Project. Nucleic Acids Res. 46 (D1), D1237–D1247. 10.1093/nar/gkx664 28985418 PMC5753376

[B96] Sheikh Q. M. Gatherer D. Reche P. A. Flower D. R. (2016). Towards the Knowledge-Based Design of Universal Influenza Epitope Ensemble Vaccines. Bioinformatics 32 (21), 3233–3239. 10.1093/bioinformatics/btw399 27402904 PMC5079473

[B97] Sirois I. Caron E. Kovalchik K. A. Wessling L. Saab F. Ma Q.. (2020). Immunopeptidomics for Dummies: Detailed Experimental Protocols and Rapid, User-Friendly Visualization of MHC I and II Ligand Datasets With Mhcvizpipe.

[B98] Small E. J. Schellhammer P. F. Higano C. S. Redfern C. H. Nemunaitis J. J. Valone F. H.. (2006). Placebo-controlled Phase III Trial of Immunologic Therapy With Sipuleucel-T (APC8015) in Patients With Metastatic, Asymptomatic Hormone Refractory Prostate Cancer. J. Clin. Oncol. 24 (19), 3089–3094. 10.1200/JCO.2005.04.5252 16809734

[B99] Song L. Song M. Camargo M. C. Van Duine J. Williams S. Chung Y.. (2021). Identification of Anti-Epstein-Barr Virus (EBV) Antibody Signature in EBV-associated Gastric Carcinoma. Gastric Cancer 1–10. 10.1007/s10120-021-01170-z 33661412 PMC8206016

[B100] Song L. Song M. Rabkin C. S. Williams S. Chung Y. Van Duine J.. (2020). Helicobacter Pylori Immunoproteomic Profiles in Gastric Cancer. J. Proteome Res. 20 (1), 409–419. 10.1021/acs.jproteome.0c00466 33108201

[B101] Strønen E. Toebes M. Kelderman S. Van Buuren M. M. Yang W. Van Rooij N.. (2016). Targeting of Cancer Neoantigens With Donor-Derived T Cell Receptor Repertoires. Science 352 (6291), 1337–1341. 10.1126/science.aaf2288 27198675

[B102] Sturm T. Sautter B. Woörner T. P. Stevanović S. Rammensee H. G. Planz O.. (2020). Mild Acid Elution and MHC Immunoaffinity Chromatography Reveal Similar Albeit Not Identical Profiles of the HLA Class I Immunopeptidome. J. Proteome Res 20, 1, 289–304. 10.1021/acs.jproteome.0c00386 PMC7786382 33141586

[B103] Tan X. Li D. Huang P. Jian X. Wan H. Wang G.. (2020). dbPepNeo: A Manually Curated Database for Human Tumor Neoantigen Peptides. Database 2020, 2020, baaa004. 10.1093/database/baaa004 PMC7043295 32090262

[B104] Ternette N. Block P. D. Sánchez-Bernabéu Á. Borthwick N. Pappalardo E. Abdul-Jawad S.. (2015). Early Kinetics of the HLA Class I-associated Peptidome of MVA. Hivconsv-Infected Cells. J. Virol. 89 (11), 5760–5771. 10.1128/JVI.03627-14 25810538 PMC4442425

[B105] Ternette N. Yang H. Partridge T. Llano A. Cedeño S. Fischer R.. (2016). Defining the HLA Class I-associated Viral Antigen Repertoire From HIV-1-infected Human Cells. Eur. J. Immunol. 46 (1), 60–69. 10.1002/eji.201545890 26467324 PMC4737398

[B106] Thurner B. Haendle I. Röder C. Dieckmann D. Keikavoussi P. Jonuleit H.. (1999). Vaccination With mage-3A1 Peptide–Pulsed Mature, Monocyte-Derived Dendritic Cells Expands Specific Cytotoxic T Cells and Induces Regression of Some Metastases in Advanced Stage IV Melanoma. J. Exp. Med. 190 (11), 1669–1678. 10.1084/jem.190.11.1669 10587357 PMC2195739

[B107] Tran N. H. Qiao R. Xin L. Chen X. Liu C. Zhang X.. (2019). Deep Learning Enables *De Novo* Peptide Sequencing From Data-Independent-Acquisition Mass Spectrometry. Nat. Methods 16 (1), 63–66. 10.1038/s41592-018-0260-3 30573815

[B108] Tran N. H. Zhang X. Xin L. Shan B. Li M. (2017). De Novo Peptide Sequencing by Deep Learning. Proc. Natl. Acad. Sci. 114 (31), 8247–8252. 10.1073/pnas.1705691114 28720701 PMC5547637

[B109] Tyanova S. Temu T. Carlson A. Sinitcyn P. Mann M. Cox J. (2015). Visualization of LC-MS/MS Proteomics Data in Maxquant. Proteomics 15 (8), 1453–1456. 10.1002/pmic.201400449 25644178 PMC5024039

[B110] Tyanova S. Temu T. Cox J. (2016). The MaxQuant Computational Platform for Mass Spectrometry-Based Shotgun Proteomics. Nat. Protoc. 11 (12), 2301. 10.1038/nprot.2016.136 27809316

[B111] Valentin F. Squizzato S. Goujon M. McWilliam H. Paern J. Lopez R. (2010). Fast and Efficient Searching of Biological Data Resources—Using EB-Eye. Briefings Bioinf. 11 (4), 375–384. 10.1093/bib/bbp065 PMC2905521 20150321

[B112] Vance R. E. Eichberg M. J. Portnoy D. A. Raulet D. H. (2017). Listening to Each Other: Infectious Disease and Cancer Immunology. Sci. Immunol. 2 (7). 10.1126/sciimmunol.aai9339 PMC5927821 28783669

[B113] Verdegaal E. M. van der Burg S. H. (2017). The Potential and Challenges of Exploiting the Vast But Dynamic Neoepitope Landscape for Immunotherapy. Front. Immunol. 8, 1113. 10.3389/fimmu.2017.01113 28959257 PMC5604073

[B114] Vita R. Mahajan S. Overton J. A. Dhanda S. K. Martini S. Cantrell J. R.. (2019). The Immune Epitope Database (IEDB): 2018 Update. Nucleic Acids Res. 47 (D1), D339–D343. 10.1093/nar/gky1006 30357391 PMC6324067

[B115] Vita R. Overton J. A. Greenbaum J. A. Ponomarenko J. Clark J. D. Cantrell J. R.. (2015). The Immune Epitope Database (IEDB) 3.0. Nucleic Acids Res. 43 (D1), D405–D412. 10.1093/nar/gku938 25300482 PMC4384014

[B116] Vizcaíno J. A. Kubiniok P. Kovalchik K. A. Ma Q. Duquette J. D. Mongrain I.. (2020). The Human Immunopeptidome Project: A Roadmap to Predict and Treat Immune Diseases. Mol. Cell. Proteomics 19 (1), 31–49. 10.1074/mcp.R119.001743 31744855 PMC6944237

[B117] Wilson E. A. Hirneise G. Singharoy A. Anderson K. S. (2021). Total Predicted MHC-I Epitope Load is Inversely Associated With Population Mortality From SARS-Cov-2. Cell Rep. Med. 2 (3), 100221. 10.1016/j.xcrm.2021.100221 33649748 PMC7904449

[B118] Zohora F. T. Rahman M. Z. Tran N. H. Xin L. Shan B. Li M. (2019). Deepiso: A Deep Learning Model for Peptide Feature Detection From LC-MS Map. Sci. Rep. 9 (1), 1–13. 10.1038/s41598-019-52954-4 31748623 PMC6868186

